# Evaluating Ruminal and Small Intestinal Morphology and Microbiota Composition of Calves Fed a *Macleaya cordata* Extract Preparation

**DOI:** 10.3390/ani13010054

**Published:** 2022-12-23

**Authors:** Janaka Wickramasinghe, Chiron J. Anderson, Can Ayhan Kaya, Patrick J. Gorden, Flavio Rodrigues Borges Ribeiro, Juliane Dohms, Sydney Rigert, Stephan Schmitz-Esser, Ranga Appuhamy

**Affiliations:** 1 Department of Animal Science, Iowa State University, Ames, IA 50011, USA; 2 Interdepartmental Microbiology Graduate Program, Iowa State University, Ames, IA 50011, USA; 3 Department of Livestock and Crop Production, Dicle University, 21280 Diyarbakir, Turkey; 4 Veterinary Diagnostic and Production Animal Medicine, Iowa State University, Ames, IA 50011, USA; 5 Phytobiotics, Futterzusatzstoffe GmbH, Wallufer Str. 10a, D-65343 Eltville, Germany

**Keywords:** crossbred calves, papillae length, phytogenic feed additive, small intestine, villus height

## Abstract

**Simple Summary:**

Mucosal morphology and microbiota composition are key components of the gut development of calves. Adverse health events during pre-weaning and diet changes at weaning can create a lasting impact on those components. Phytogenic feed additives such as the *Macleaya cordata* plant extract preparations (MCE) improved the gut morphology and microbiota composition of pigs and poultry in previous studies. In this study, MCE was fed to dairy × beef crossbred calves before (6 to 45 d) and after weaning (45 to 90 d). The cumulative impact on the ruminal and small intestinal morphology and microbiota composition were evaluated at 95 d of age. Despite the minor changes in ruminal and intestinal microbial populations, MCE increased the rumen papillae length and villus height: crypt depth in the small intestine, suggesting the potential to enhance gut health and development.

**Abstract:**

The objective was to determine the impact of feeding MCE on ruminal and intestinal morphology and microbiota composition of calves. A total of 10 male and 10 female crossbred (dairy × beef) calves (6 d of age) were assigned randomly to control (CTL; *n* = 10) or MCE-supplemented (TRT; *n* = 10) groups. The MCE was fed in the milk replacer and top-dressed on the calf starter during pre-weaning (6 to 49 d) and post-weaning (50 to 95 d) periods, respectively. Calves were slaughtered at 95 d to collect rumen and intestinal samples to determine volatile fatty acid (VFA) profile, mucosal morphology, and microbiota composition. The effects of MCE were analyzed by accounting for the sex and breed effects. Feeding MCE increased rumen papillae length (*p* = 0.010) and intestinal villus height: crypt depth (*p* < 0.030) compared to CTL but did not affect rumen VFA profile. The TRT had a negligible impact on microbial community composition in both the rumen and the jejunum. In conclusion, feeding MCE from birth through weaning can improve ruminal and small intestinal mucosa development of calves despite the negligible microbiota composition changes observed post-weaning.

## 1. Introduction

*Macleaya cordata*, commonly called plume poppy, is a plant native to China and Japan, where it is used for medicinal purposes. *M. cordata* belongs to the tribe *Chelidonieae* of the family *Papaveraceae*, which produces colored latex rich in alkaloids [1]. Among those alkaloids, sanguinarine and chelerythrine are predominant and possess antimicrobial and anti-inflammatory properties [2] that have been shown to modulate gut microbiota and improve small intestinal morphology to enhance growth [3]. Therefore, plant extracts rich in those alkaloids can substitute antibiotics aiming to promote the growth of young animals [3,4,5]. In support, dietary supplementations of MCE enhanced intestinal morphology (e.g., longer villi) and increased the average daily gain (ADG) of pigs and chickens [3,6,7]. Similar effects could be hypothesized for calves, as Lima et al. [8] demonstrated that MCE improved the ruminal epithelial development in lambs. Dietary MCE has been also shown to affect VFA profiles in the cecum of broiler chickens [9], suggesting an ability of MCE to modulate gut microbiota composition, an important component of gut development. Liu et al. [10] demonstrated that dietary MCE increased the abundance of beneficial bacteria such as *Lactobacillus* and *Bifidobacterium* in the caecum, whereas it decreased the abundance of pathogenic bacteria such as *E. coli* and *Salmonella* in the ileum of piglets. The impact of MCE on the gut microbiota composition of cattle is unknown. Given the unique complexity of the ruminal gastrointestinal tract, the findings of monogastric animals cannot be extrapolated directly to cattle. On the other hand, ruminal VFA profiles can reflect the changes in ruminal bacteria composition. For instance, increased propionate and butyrate concentrations were associated with an increased abundance of *Lactobacillaceae* in the rumen of calves [11]. High propionate and butyrate concentrations in the rumen can stimulate ruminal papillae development and feed intake [12,13]. Interestingly, ruminal papillae development can be positively associated with the small intestinal mucosal development of calves [14]. Calf diarrhea and weaning stresses can impede gastrointestinal development of calves, and nutritional interventions are key to ameliorate those effects [15,16]. Hence, it was hypothesized that feeding MCE from birth through weaning would improve the ruminal and small intestinal development of calves. The study objective was to determine the effects of feeding MCE in milk replacers (pre-weaning) and in starter feed (post-weaning) on the mucosal morphology and microbiota composition in the rumen and the small intestine of weaned calves.

## 2. Materials and Methods

### 2.1. Animals and Treatments

All animal-related procedures were conducted under the approval of the Animal Care and Use Committee of Iowa State University (IACUC-19-204). The experiment was conducted at the Dairy Research and Teaching Farm at Iowa State University (Ames, IA). Forty dairy (Holstein) × beef (Angus or Simmental) crossbred calves (20 male and 20 female) at 5 d of age were balanced for sex, and assigned to four doses (0, 2, 5 and 10 g/d pre-weaning, and 0, 4, 10 and 20 g/d post-weaning) of an MCE preparation (Sangrovit^®^, Phytobiotics Futterzusatzstoffe GmbH, Eltville, Germany). Dose-dependent effects of MCE on feed intake, growth, and safety are reported in Matulka et al. [17]. Given the linear increase in calf starter intake with MCE dose [17] and the positive relationship between starter intake and gut development of calves [18], the gut morphology and microbiota analyses were performed only on calves in 0 g/d (CTL) and the highest dose (10 calves per treatment group). The latter is referred to as the MCE-feeding group (TRT) in this paper, reporting only the results of those two analyses. Further, a statistical power analysis (power = 80% and α = 0.05) using the data from Alves Costa et al. [19] revealed that the sample size = 10 could be adequate to capture 10 and 15% change from average rumen papillae length and villus height of crossbred calves, respectively. The sample size = 10 was assumed to be adequate to capture treatment differences in gut microbiota community composition, as Wang et al. [20] captured such differences in rats in response to feeding an MCE with a sample size = 6.

The baseline (5 d of age) serum total protein by refractometer (mean = 7.30% and SD = 0.77%) and the body weight (mean = 45.3 kg and SD = 6.5 kg) were similar between CTL and TRT. Calves were housed individually in outdoor calf hutches (2.4 m × 1.4 m × 1.3 m) bedded with straw throughout the study. The MCE preparation (0 or 10 g/d) was fed in milk replacer during the pre-weaning period (up to 49 d). The feeding rate of MCE was doubled (20 g/d) to be in line with the dry matter intake increase [16] and fed with a calf starter during the post-weaning period (50 to 95 d). The daily dose of MCE was top-dressed on a calf starter by mixing with the topmost layer (about 1–2” deep) of the starter in the morning (0700 h). When needed, the starter buckets were refilled in the afternoon by placing the morning feed on top of the new feed to ensure complete consumption of the MCE dose. The composition of the milk replacer (Land O’Lakes^®^, St. Louis, MI, USA) and the calf starter (Farmers Win Coop^®^, Houston, MN, USA) is given in Table 1.

### 2.2. Feeding and General Management

#### 2.2.1. Pre-Weaning

A total of 6.0 L of liquid milk replacer (12% solids) was fed daily at two feedings (3.0 L per feeding at 0730 and 1930 h) until the beginning of weaning. One-half of the MCE dose was hand-mixed with 3.6 kg of dry milk replacer, which was then reconstituted with 27.0 kg of lukewarm water (40 to 45 °C) to prepare the liquid milk replacer at a single feeding. In addition to the MCE dose, 10 g of a coccidiostat containing decoquinate (Zoetis, Parsippany-Troy Hills, NJ, USA) was added to dry milk replacer of both CTL and TRT until calves were 28 d of age. The weaning process was started at 42 d of age by eliminating the morning milk replacer feeding. All the calves then received only 3.0 kg/d liquid milk replacer until weaned completely at 49 d of age. For TRT calves, the total MCE dose was fed in 3.0 kg/d liquid milk replacer during weaning (d 42 to 49). The weights of liquid milk replacer offered to and left over by individual calves were recorded daily. 

#### 2.2.2. Post-Weaning

The MCE dose was top-dressed on calf starter (110% of previous day intake) and offered to calves at 0700 h during the post-weaning period. To avoid losses due to wind and animal activities (e.g., coughing and exhaling), the dose was gently mixed into the topmost layer (about one inch deep) of the calf starter before offering to the animals. The amounts of calf starter offered, and leftover were recorded daily. Multiple batches of starter feed were used during the study. Representative samples of each batch were analyzed for nutrient composition using the standard procedures (Cumberland Valley Analytical Services, Waynesboro, PA, USA). The average composition is presented in Table 1.

### 2.3. Ruminal and Intestinal Sample Collection

At the end of the study (95 d of age), calves were slaughtered to collect digesta samples and segments of the rumen and the small intestine for microbiota analysis and morphometry analysis, respectively. The slaughtering involved stunning with a captive bolt pistol followed by immediate exsanguination at the livestock infectious disease isolation facility of Iowa State University (Ames, IA, USA). About 300 g of rumen content of the ventral sac and about 50 g of digesta from the jejunum were retrieved and placed immediately in dry ice until transfer to a −80 °C freezer. The jejunum was specifically chosen for the microbiome analysis (described below), as both digesta and mucosa of the jejunum were shown to have a greater and similar density of bacteria (numbers per gram wet weight) compared to those of the duodenum and ileum in cattle, respectively [21]. Additionally, 4.0–6.0 cm^2^ segments of the rumen (ventral sac), and 2–3 cm long segments of the duodenum, jejunum (15 and 80 cm distal to pyloric sphincter, respectively), and ileum (15 cm proximal to ileocecal junction) were obtained. All samples were flushed with saline to remove any digesta and were fixed in 10% neutral-buffered formaldehyde (Shandon Formal-Fixx^®^, Thermo Scientific, Waltham, MA, USA) for the morphometric analysis described below. 

### 2.4. Morphometry Analysis of Rumen and Small Intestinal Mucosa

The rumen, duodenum, jejunum, and ileum samples were fixed in 10% neutral buffered formalin and submitted for tissue sectioning and imaging at the Veterinary Diagnostic Laboratory at Iowa State University (Ames, IA, USA). After trimming into plastic cassettes, specimens were routinely processed overnight (Tissue-Tek VIP 6, Sakura Finetek, Tokyo, Japan), paraffin-embedded, and cut to 4-micron-thick sections on an automated microtome (Leica Microtome RM2255, Leica Biosystems, Deer Park, IL USA). Tissue sections were placed on glass slides (Surgipath Snowcoat clipped corner slides, Leica Biosystems, Deer Park, IL USA) and stained with hematoxylin and eosin on an automated stainer (Tissue-Tek Prisma Autostainer, Sakura Finetek, Tokyo, Japan) and glass coverslipped (G2 Automated Glass Coverslipper, Sakura Finetek, Tokyo, Japan). The images of tissue sections were obtained at 10× magnification using an Olympus DP73 camera mounted onto an Olympus B53 microscope (Olympus Corporation, Shinjuku City, Tokyo, Japan). Papillae length and width, villus height, and crypt depth in three non-overlapping fields of each tissue cross-section were measured using the ImageJ software (National Institutes of Health, Bethesda, MD, USA). The arbitrary units obtained from the ImageJ software were converted to mm by using a reference image obtained from the microscope. The length and width of rumen papillae and villus height and crypt depth of the small intestine were averaged across the three fields of each image representing a sample from an individual animal. The villus height was divided by corresponding crypt depth to calculate villus height to crypt depth ratio.

### 2.5. Rumen VFA Analysis

Rumen content samples were thawed on ice and squeezed through four layers of cheesecloths to collect rumen fluid (~15 mL per sample). All solids and approximately 5 mL of rumen fluid were stored again at −80 °C for the microbiota composition analyses. The remaining liquid was centrifuged at 4 °C and 9000× *g* for 15 min. Then, 5 mL of the supernatant was mixed with 1.0 mL of 25% metaphosphoric acid and centrifuged again for 10 min at 4000× *g* at room temperature, and the supernatant was separated. Solutions of acetate, propionate, butyrate, isobutyrate, valerate, and isovalerate, each at 1:1, 1:2, 1:4, and 1:6 dilutions were prepared using deionized water and used as the standards. 100 µL of 2-ethyl butyric acid was added as an internal standard to all samples as well as the standards. Concentrations of VFA in samples were analyzed using Varian CP-3800 Gas Chromatograph (Varian Medical Systems, Palo Alto, CA, USA).

### 2.6. DNA Extraction and Sequencing

DNA was extracted from 0.25 g of each rumen liquid and solid and jejunal digesta and mucosa samples using the Qiagen DNeasy Powersoil Powerlyzer Kit^®^ (Qiagen Sciences Inc., Germantown, MD, USA) according to the protocol provided by the manufacturer. The process included a bead-beating step for one minute at 22 °C using a Fisherbrand^TM^ Bead Mil 24 Homogenizer (Fisher Scientific, Portsmouth, NH, USA) for mechanical lysis of the cells. The concentration of DNA was quantified using a NanoDrop 2000 spectrophotometer (Thermo Scientific, Waltham, MA, USA) and the concentrations were adjusted to 25 to 50 ng of DNA/µL using PCR-grade water. All samples were stored at −80 °C until being submitted to Iowa State University DNA Facility (Ames, IA, USA) for sequencing.

DNA sequencing was conducted at the Iowa State University DNA Facility using an Illumina MiSeq platform (Illumina, Inc., San Diego, CA, USA) and a protocol designed to amplify the 16S rRNA genes of bacteria and archaea. Template DNA from each sample (one replicate per sample) was amplified using the Platinum^TM^ Hot Start PCR Master Mix (Thermo Fisher Scientific, Waltham, MA, USA) with the 515F forward-barcoded primer (GTGYCAGCMGCCGCGGTAA) [22], and the 806R reverse primer (GGACTACNVGGGTWTCTAAT) [23]. All samples underwent an initial denaturation step at 94 °C for 2 min, followed by denaturation at 94 °C for 45 s, annealing at 50 °C for 20 s, and extension at 72 °C for 90 s. Those steps were repeated 35 times. Amplicons from each sample were run on an agarose gel with an expected band size ranging from 300 to 350 bp. Amplicon pools were cleaned using Qiagen’s QIAquick PCR Purification Kit (Qiagen Sciences Inc., Germantown, MD, USA). Raw sequencing data from each sample location was analyzed separately using the mothur software (v1.43.0) [24]. The number of raw reads per sample varied from 13,742 to 473,268. All reads were subjected to quality control using the “*screen.seqs*” command to remove contigs with ambiguous bases, homopolymers greater than eight bases in length, as well as contigs below a minimum length of 252 bases. Sequences were aligned against the SILVA SSU database (v138) and sequences outside the target variable region were removed [25]. Possible chimeric sequences were detected and removed using the “chimera.vsearch” command and the SILVA.gold reference database provided by the mothur website (https://mothur.org/wiki/silva_reference_files/, accessed on 15 January 2021). The remaining 9,482,307 high-quality sequences were clustered into de novo operational taxonomic units (OTU) with a 99% similarity threshold using the “cluster.split” command. The OTU abundance data were imported into R v3.6.2 for further processing. OTUs represented by fewer than 10 reads were removed, resulting in a total of 2606 OTUs in jejunal content, 1984 OTUs in jejunal mucosa, 2761 OTUs in rumen solid and 2895 OTUs in rumen liquid samples. Alpha diversity parameters including Chao 1, Shannon, Simpson, and inverse-Simpson indices were determined by using the Phyloseq R package (v1.32, https://github.com/joey711/phyloseq, accessed on 15 January 2021) [26]. Beta diversity was analyzed by using the Adonis and beta-dispersion tests with the R package vegan. Genera abundance heatmaps were generated with ggplot2 (v3.2.1, https://CRAN.R-project.org/package=ggplot2, accessed on 15 January 2021) using a color palette from the viridis package (v0.5.1, https://CRAN.R-project.org/package=viridis, accessed on 15 January 2021) in the R scripting language (The R Foundation, Indianapolis, IN, USA).

### 2.7. Statistical Analysis

The MCE dose effect on gut morphology and rumen VFA measurements, alpha-diversity indices of gut microbiota composition, and relative abundance of 50 most abundant genera or 100 most abundant OTU were analyzed by using the GLM procedure of SAS 9.4 (SAS Institute Inc., Cary, NC, USA) with the following model:

Y_ijkl_ = μ + T_i_ + S_j_ + B_k_ + (T × S)_ij_ + e_ijkl_ (1)

where Y_ijklm_ = the response variable of interest, μ = overall mean, T_i_ = treatment effect (i = CTL and TRT), S_j_ = effect of sex, B_k_ = effect of breed, (T × S)_ij_ = interaction effect between treatment and sex, and e_ijklm_ = the residual error. When analyzing the villus height, crypt depth, and villus height to crypt depth ratio, the fixed effect of the region (duodenum, jejunum, or ileum) was included in the model, and the treatment effect within each region was assessed with the slice option of the SAS. The significance of treatment effects on the microbiota abundance was assessed by adjusting the raw *p*-values for false discovery rates (FDR) and by calculating FDR-adjusted *p*-values (*q*-values) according to Benjamini and Hochberg [27].

## 3. Results and Discussion

### 3.1. Effects of MCE on Gut Morphology and Rumen VFA

The effects of feeding MCE on rumen papillae and small intestinal morphology are presented in Table 2. In alignment with weaned piglets [28], TRT increased villus height and villus height: crypt depth in duodenum and ileum (*p* < 0.070), and villus height: crypt depth in the jejunum (*p* = 0.022) of the small intestine. Additionally, TRT increased crypt depth in the jejunum compared to CTL (*p* = 0.033). The data did not support an effect of sex (*p* = 0.250), sex × treatment (*p* = 0.130), or breed (*p* = 0.760) on any intestinal morphology parameters across the duodenum, jejunum, and ileum. Taller villi represent increased digestive and absorptive capacities, whereas deeper crypts can indicate inflammations in the intestinal epithelium [29]. Moreover, high villus height to crypt depth ratios can be a positive indicator of intestinal barrier function, reducing pathogen and toxin transfer from intestinal digesta to the bloodstream [29,30]. Feeding MCE also increased papillae length in the rumen (*p* = 0.013), while papillae width remained similar between CTL and TRT suggesting an elevated capacity for VFA absorption. In support, Lima et al. [8] demonstrated an increased surface area of rumen papillae in MCE-fed sheep. The data did not support an effect of sex (*p* = 0.649) or sex × treatment (*p* = 0.197) on papillae length. The papillae width of male calves was greater than that of female calves (535 vs. 485 mm, *p* = 0.002). Nevertheless, the consistent responses of both rumen papillae and intestinal villi to MCE agreed with Górka et al. [31], demonstrating simultaneous responses of the rumen and small intestine development to nutritional interventions in dairy calves. The improvements in the ruminal and intestinal morphology can be a result of sanguinarine and chelerythrine in MCE that inhibited the programmed cell death of intestinal mucosa of rats [32] and enhanced the recovery from enteritis in broiler chicks [33]. Given the high risk of intestinal damage and the responsiveness of the intestine to dietary bioactive compounds in pre-weaning calves [34], the improved intestinal morphology observed post-weaning could be a result of a response to MCE pre-weaning. A comprehensive evaluation representing both pre- and post-weaning stages could better explain the effects of MCE on the gut morphology of calves. 

The effects of MCE on VFA concentrations and the molar percentages in the rumen are presented in Table 3. The MCE in the diet did not affect (*p* > 0.100) the individual or total VFA concentrations, which are net outcomes of VFA production and VFA absorption in the rumen. High molar percentages of butyrate and propionate have been shown to stimulate the morphological and metabolic developments of the rumen [14]. Due to the greater VFA absorption potential indicated by longer papillae of TRT than CTL, one could speculate an increased VFA production in response to feeding MCE. The sum of propionate and butyrate molar percentages increased (25%) only numerically in TRT compared to CTL (*p* = 0.154). MCE did not affect the molar percentages of acetate, valerate, isovalerate, or isobutyrate (*p* > 0.550). On the contrary, Aguilar-Hernandez et al. [35] observed increased molar percentage of acetate and decreased molar percentages of valerate and isovalerate in the rumen of steers fed an MCE preparation. Differences in the basal diet composition (calf starter vs. total mixed ration), MCE feeding duration (90 vs. 21 d), and MCE feeding frequency (once vs. twice per d) between two studies might explain the discrepancy. Overall, the VFA data do not corroborate the MCE-induced rumen papillae development. Potentially, the current sampling time (13 wk) missed the fermentation responses that preceded the papillae development response to feeding MCE. van Niekerk et al. [36] observed significant changes in rumen VFA profiles at an earlier age (7–9 wk of age) than the age they could capture papillae development changes (12 wk of age) in calves weaned at 6 wk of age. Moreover, the present data did not support an effect of sex (*p* = 0.330), sex × treatment (*p* = 0.120), or breed (*p* = 0.270) on concentration or molar percentage of VFA in the rumen of post-weaned calves.

### 3.2. Digesta- and Mucosa-Associated Microbiota in the Jejunum of Weaned Calves

**Figure 1:**
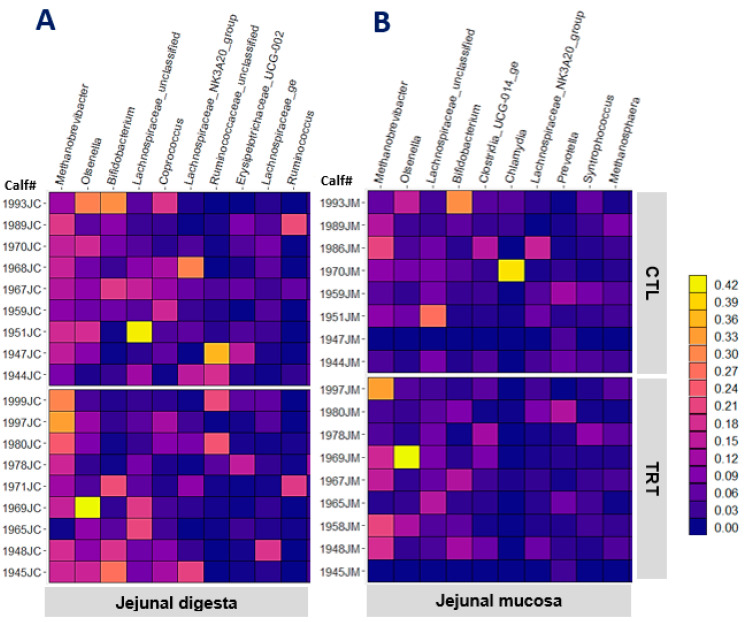
The relative abundance of the 10 most abundant genera in jejunal digesta (**A**) and jejunal mucosa (**B**) of weaned calves (95 d old) in control (CTL) and MCE-fed (TRT) groups.

Feeding MCE did not affect any of the alpha diversity indices of digesta- or mucosa-associated microbiota in the jejunal digesta or jejunal mucosa (*p* > 0.220). The heat map in Figure 1 depicts the relative abundance of the 10 most abundant genera in jejunal mucosa and jejunal digesta. *Methanobrevibacter*, *Olsenella*, *Lachnospiraceae_UC*, *Bifidobacterium_UC*, and *Clostridia_UCG-014_ge* were the most abundant genera common across the digesta and mucosa of the jejunum. The genera previously reported to be the most abundant in the intestinal mucosa of pre-weaned calves were different from that of the weaned calves in this study [21]. This discrepancy corroborates the notion that diet change at weaning is a predominant factor shaping the gut microbiota composition of calves [34,37]. Table 4 presents the top genera having different relative abundance between CTL and TRT calves according to raw *p*-values from the statistical analysis (*p* ≤ 0.050). Feeding MCE decreased the abundance of *Erysipelotrichaceae_UC* in the digesta (*p* = 0.016) and *Lachnospiraceae_NK3A20_group* in the mucosa (*p* = 0.037). The high FDR-adjusted *p*-values (*q* > 0.80).), however, point out a lack of strong evidence to support those effects. Table 5 presents OTUs among the 100 most abundant, showing different relative abundances between CTL and TRT calves based on the raw *p*-values (*p* ≤ 0.050). The abundance of two digesta OTUs closely related to *Eubacterium pyruvativoran* (*p* = 0.025), and *Gastranaerophilales_UC* (*p* = 0.045) seem to be different between TRT and CTL calves. The abundance of four mucosa-associated OTUs also seem to be different between CTL and TRT calves (*p* < 0.050). Nevertheless, the high FDR-adjusted *p*-values (*q* > 0.550) signify the lack of strong evidence to support an effect of feeding MCE from birth on the microbiota composition of jejunal mucosa of weaned calves. The digesta and mucosa samples from a greater number of animals at multiple time points (e.g., both pre-weaning and post-weaning) could have increased the likelihood of deriving robust conclusions about the MCE effects. Nonetheless, studies such as this one investigating the microbiota composition of the calf small intestine are limited in literature. Most of the available studies rely on fecal samples to describe intestinal microbiota composition. 

### 3.3. Liquid- and Solid-Associated Microbiota in the Rumen of Weaned Calves

**Figure 2:**
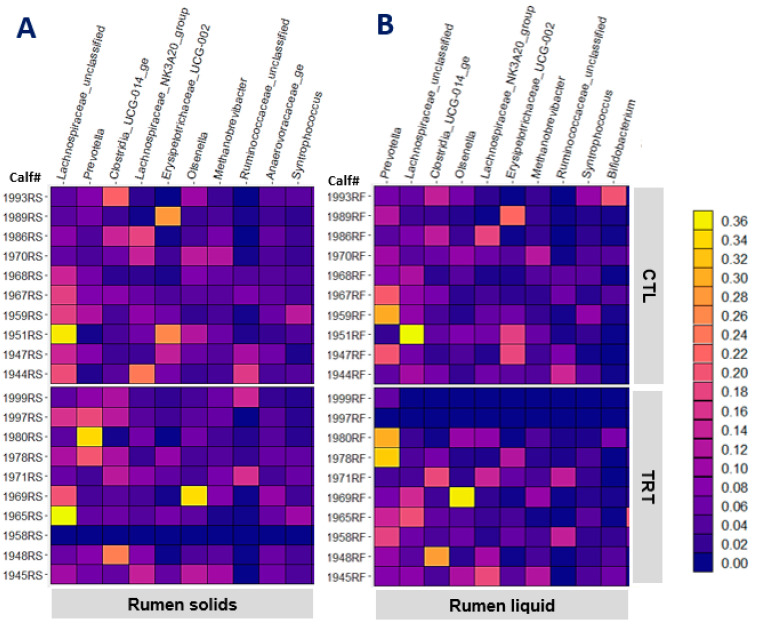
The relative abundance of the 10 most abundant genera in rumen solids (**A**) and rumen liquid (**B**) of weaned calves (95 d old) in control (CTL) and MCE-fed (TRT) groups.

Feeding MCE did not affect any of the alpha diversity indices of liquid- or solid-associated taxa in the rumen (*p* > 0.230). The heat maps in Figure 2A,B depict the relative abundance of the 10 most abundant genera in rumen solid and liquid, respectively. The most abundant jejunal genera, *Lachnospiraceae_UC, Lachnospiraceae_NK3A20_group, Olsenella*, and *Methanobrivibacter* were also among the most abundant bacteria in the rumen. Of the 50 most abundant genera in each solid or liquid-associated microbiota, feeding MCE was confirmed with strong evidence (*p* = 0.002 and *q* = 0.110, Table 4) to have an impact on the abundance of only the genus *Lachnospira* in rumen solid. Feeding MCE since birth decreased the abundance of *Lachnospira* in the rumen solid of weaned calves. The low abundance of *Lachnospira* could be beneficial as Huang et al. [38] demonstrated a lower abundance of *Lachnospira* in the rumen of cows with high feed efficiency than those with low feed efficiency. Based on the raw *p*-values, the abundance of OTU60 (rumen solid) and OTU89 (rumen liquid) closely related to *Blautia caecimuris* decreased in response to feeding MCE. Regardless of the *q*-values not supporting robust conclusions (*q* ≥ 0.390), the abundance reduction consistent in both rumen solid and liquid corroborates an inhibitory effect of MCE on *Blautia caecimuris* in the rumen.

## 4. Conclusions

Feeding MCE from birth through weaning increased papillae length in the ventral sac of the rumen, villus height in the duodenum and ileum, and villus height: crypt depth ratio in the duodenum, jejunum, and ileum of weaned calves (13 wk of age). Feeding MCE, however, did not change the rumen VFA profile and had negligible effects on microbial community composition in the rumen and the jejunum of those calves. Future studies with periodic sampling covering both pre-weaning and post-weaning stages are warranted to better understand the effects of MCE on the gut development of calves.

## Figures and Tables

**Table 1:** Ingredient and nutrient composition of milk replacer and calf starter.

Item	Content
***Milk Replacer***	
**Nutrient composition (% of DM unless otherwise specified) ^1^**	
Crude protein	22.00
Crude fat	20.00
Crude fiber	0.15
Calcium	1.00
Phosphorus	0.70
Vitamin A	9071.85 IU/kg
Vitamin D3	2267.96 IU/kg
Vitamin E	45.36 IU/kg
***Calf Starter***	
**Ingredient composition (% of as-fed) ^1^**	
Whole corn, shelled	54.0
Whole oats	7.5
Heifer pellet ^2^	35.0
Molasses, liquid	2.5
Corn oil	1.0
**Nutrient composition (% of DM, unless otherwise specified) ^3^**	
Dry matter, % of as-fed	87.9
Crude protein	20.2
Neutral detergent fiber	15.2
Acid detergent fiber	7.3
Starch	41.5
Non-structural carbohydrates	54.0
Crude fat	4.2
Ash	6.3

^1^ Reported by the manufacturer; ^2^ did not contain antimicrobial, phytogenic, or appetite-enhancing additives, and composed of 54.6% soybean meal, 11.7% wheat middlings, 10.0% wheat red dog, 8.4% pork meat and bone meal, 5.3% beet pulp, 4.0% molasses, and 6.0% mineral and vitamin premix (as-fed basis); ^3^ measured in a certified laboratory; DM = dry matter; IU = International unit.

**Table 2:** The least-square means and associated standard error of the mean (SEM) for parameters representing gastrointestinal morphology in control (CTL) and MCE-fed calves (TRT).

Variable	CTL	TRT	SEM	*p*-Value
**Rumen**				
Papillae length, mm	2.68	3.45	0.16	0.013
Papillae width, mm	0.51	0.51	0.01	0.871
**Duodenum**				
Villus height, mm	0.45	0.57	0.03	0.010
Crypt depth, mm	0.48	0.47	0.03	0.614
Villus height: crypt depth	1.02	1.40	0.11	0.027
**Jejunum**				
Villus height, mm	0.48	0.52	0.03	0.128
Crypt depth, mm	0.55	0.47	0.03	0.033
Villus height: crypt depth	0.95	1.34	0.11	0.022
**Ileum**				
Villus height, mm	0.52	0.62	0.03	0.028
Crypt depth, mm	0.48	0.47	0.03	0.566
Villus height: crypt depth	1.19	1.34	0.11	0.065

**Table 3:** The least-square means and associated standard error of the mean (SEM) for volatile fatty acid (VFA) concentrations and molar percentages of VFA in the rumen of control (CTL) and MCE-fed calves (TRT).

Variable	CTL	TRT	SEM	*p*-Value
**VFA Concentration, mM**				
Acetate	81.50	75.50	7.60	0.541
Propionate	32.08	31.62	3.40	0.918
Butyrate	12.49	11.77	2.49	0.823
Valerate	3.97	3.92	0.50	0.940
Isovalerate	7.53	4.15	1.72	0.147
Isobutyrate	0.84	0.89	0.06	0.490
Total VFA	138.43	127.82	9.00	0.374
**VFA molar percentage**				
Acetate	59.68	59.73	3.06	0.990
Propionate	23.31	28.25	3.66	0.308
Butyrate	9.01	14.68	3.56	0.232
Propionate + Butyrate	32.31	42.93	5.53	0.154
Valerate	3.70	4.60	1.25	0.580
Isovalerate	7.61	5.45	3.14	0.600
Isobutyrate	0.83	1.12	0.37	0.551
Propionate: acetate	0.40	0.49	0.07	0.323

**Table 4:** Genera within the 50 most abundant showing differences (*p* ≤ 0.05) between control (CTL) and MCE-fed (TRT) calves.

Genera	Abundance *		SEM	*p*-Value	*q*-Value
	CTL	TRT			
**Jejunal digesta**					
*Erysipelotrichaceae_UC*	0.016	0.003	0.004	0.016	0.805
**Jejunal mucosa**					
*Lachnospiraceae_NK3A20_group*	0.045	0.010	0.010	0.037	0.954
**Rumen solid**					
*Lachnospira*	0.003	0.001	0.001	0.002	0.110
*NK4A214_group*	0.003	0.024	0.007	0.052	0.822
**Rumen liquid**					
*Selenomonadaceae_UC*	0.012	0.001	0.003	0.026	0.714

* The least-square means of the relative abundance.

**Table 5:** Operational taxonomic units (OTU) within the 100 most abundant showing differences (*p* ≤ 0.05) between control (CTL) and MCE-fed (TRT) calves.

OTUs	Abundance *		SEM	*p*-Value	*q*-Value
	CTL	TRT			
**Jejunal digesta**					
*Eubacterium pyruvativoran* (OTU23)	0.0040	0.0149	0.0032	0.025	0.794
*Gastranaerophilales_UC* (OTU75)	0.0028	0.0001	0.0009	0.045	0.794
**Jejunal mucosa**					
*Ruminococcus lactaris* (OTU05)	0.0508	0.0080	0.0115	0.041	0.782
*Methanobrevibacter boviskoreani* (OTU13)	0.0440	0.0129	0.0175	0.047	0.782
*Blautia caecimuris* (OTU74)	0.0010	0.0054	0.0009	0.006	0.570
*Bifidobacterium faecale* (OTU90)	0.0001	0.0044	0.0011	0.029	0.782
**Rumen solid**					
*Blautia caecimuris* (OTU60)	0.0071	0.0001	0.0012	0.004	0.390
**Rumen liquid**					
*Kineothrix alysoides* (OTU29)	0.0151	0.0035	0.0033	0.021	0.812
*Blautia caecimuris* (OTU89)	0.0050	0.0002	0.0016	0.018	0.812

* Least-square means of the relative abundance.

## Data Availability

All datasets collected and analyzed during the current study are available from the corresponding author on fair request.
